# P-134. Staphylococcal versus Enterococcal Endocarditis: Lesser of the Two Evils?

**DOI:** 10.1093/ofid/ofaf695.361

**Published:** 2026-01-11

**Authors:** Saira P Iqbal, May Thet Hmu Tun, Khin Htet Htet Soe, Sumitra Paudel, Khant Kaung Htet Lwin, Krishna Vamsy Polepalli, Prarath Roshni, Khine Nyein Chan, Monica Ghitan, Edward Chapnick, Jessica Chung, Yu Shia Lin

**Affiliations:** Maimonides Health, Brooklyn, NY; Maimonides Health, Brooklyn, NY; Maimonides Health, Brooklyn, NY; Maimonides Medical Center, Brooklyn, New York; Maimonides Medical Center, Brooklyn, New York; Maimonides Medical Center, Brooklyn, New York; Maimonides Medical Center, Brooklyn, New York; Maimonides Medical Center, Brooklyn, New York; Maimonides Medical Center, Brooklyn, New York; Maimonides Medical Center, Brooklyn, New York; Maimonides Health, Brooklyn, NY; Maimonides Medical Center, Brooklyn, New York

## Abstract

**Background:**

Infective endocarditis (IE) was once considered a relatively uncommon, yet life-threatening illness resulting from the invasion of cardiac tissue by circulating microorganisms. However, according to the Global Burden of Disease (GBD) Study, the global incidence of IE has increased by 40% over the past decade. Many IE cases could be attributable to recent advances in cardiac interventions, such as minimally invasive transcatheter valve replacements. *Staphylococcus* and *Enterococcus* species are the two most common pathogens responsible for most IE cases. This study aims to compare the clinical characteristics and outcomes of IE caused by these pathogens.

**Table 1:**
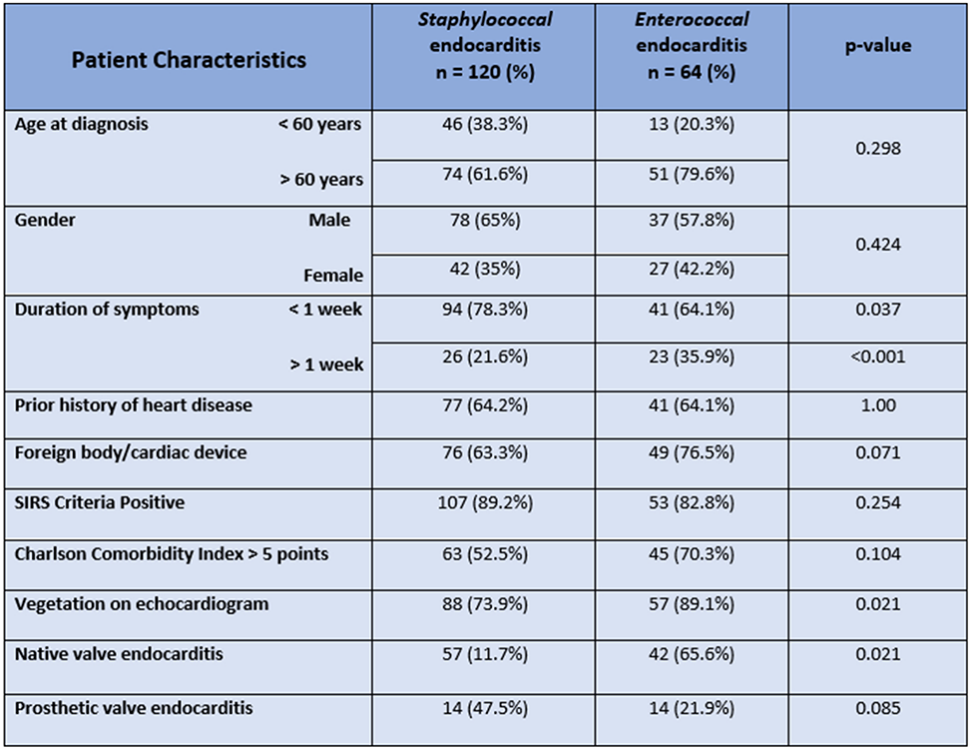
Demographics and Baseline Characteristics of SIE vs. EIE

**Table 2:**
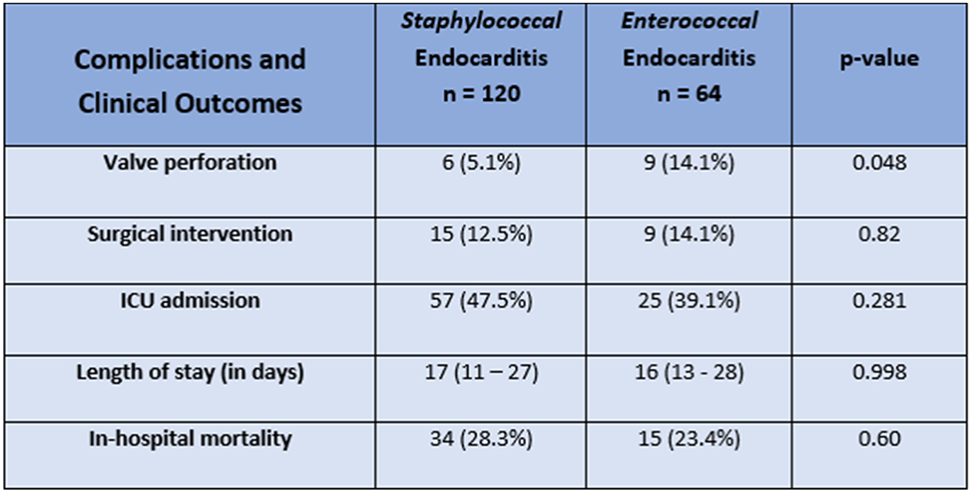
Complications and Clinical Outcomes of SIE vs EIE

**Methods:**

A retrospective chart analysis was conducted for patients admitted at a tertiary care hospital from January 1, 2016, to June 30, 2024, who had a presumptive or definite diagnosis of IE based on the 2023 Duke-ISCVID IE criteria and received at least 72 hours of antibiotics. They were classified into Staphylococcal IE (SIE) and Enterococcal IE (EIE) groups. After initial screening, 184 cases were included for chart review.

**Figure 1:**
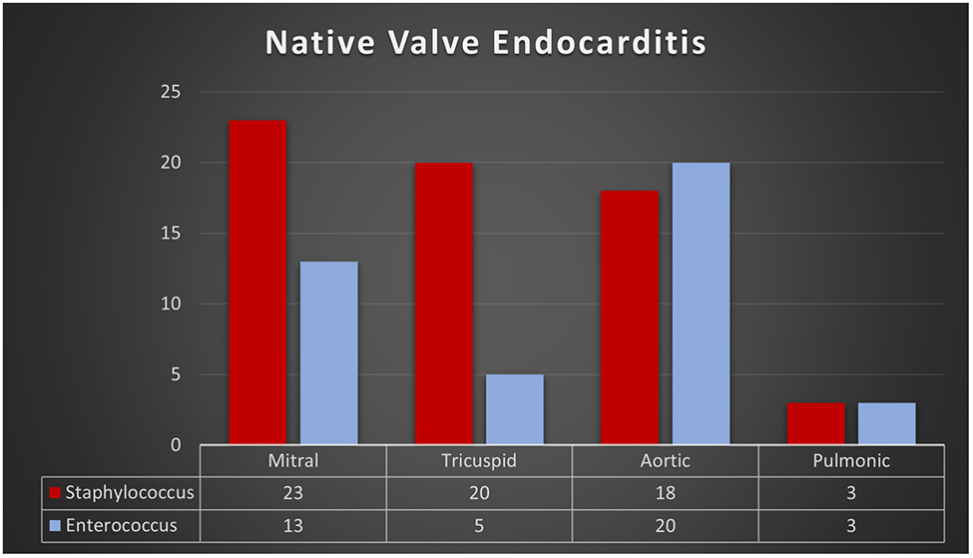
Native Valve Endocarditis caused by Staphylococcus and Enterococcus spp.

**Figure 2:**
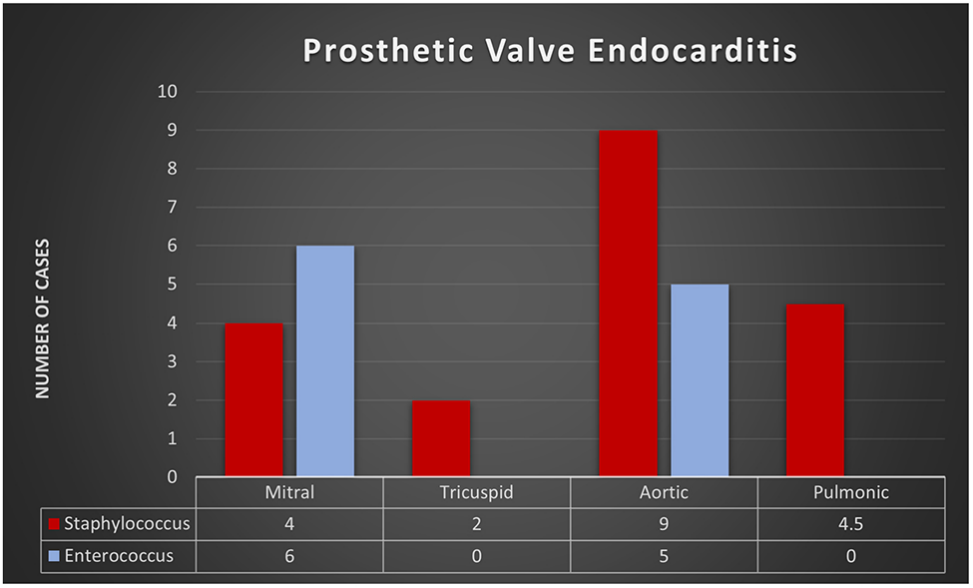
Prosthetic Valve Endocarditis caused by Staphylococcus and Enterococcus spp.

**Results:**

Of the 184 patients, 120 (65%) had SIE, while 64 (35%) had EIE. EIE patients were older and had higher Charlson Comorbidity Index (CCI) > 5 compared to the SIE group, as detailed in Table 1. Notably, 36% of EIE cases had a longer symptom duration prior to their index admission, compared to 20% of those with SIE. Echocardiograms revealed that vegetation was detected in 89% of EIE cases, compared to 73.9% of SIE cases (*p = 0.021*). Native valve involvement was higher in the EIE than in the SIE group (65.6% vs. 47.5%, *p = 0.021*). Additionally, native aortic valves were significantly more affected by enterococcus (*p = 0.002*), and the EIE group showed a higher risk of valve perforation (*p = 0.04*). Both groups had comparable complications and clinical outcomes, as shown in Table 2.

**Conclusion:**

This study highlights distinct clinical differences between EIE and SIE.

EIE often affects older patients and can have a subacute presentation. This may lead to delayed diagnosis with higher rates of vegetation detection and valve perforation, as observed in our study. These findings underscore the importance of maintaining clinical suspicion and ensuring early diagnosis and targeted antimicrobial management for EIE patients.

**Disclosures:**

All Authors: No reported disclosures

